# Supplementary figures and images for: GOLPH3 Participates in Mitochondrial Fission and Is Necessary to Sustain Bioenergetic Function in MDA-MB-231 Breast Cancer Cells
Source: Cells. 2024 Feb 8;13(4):316. doi: 10.3390/cells13040316 (PMC10887169; doi:10.3390/cells13040316)

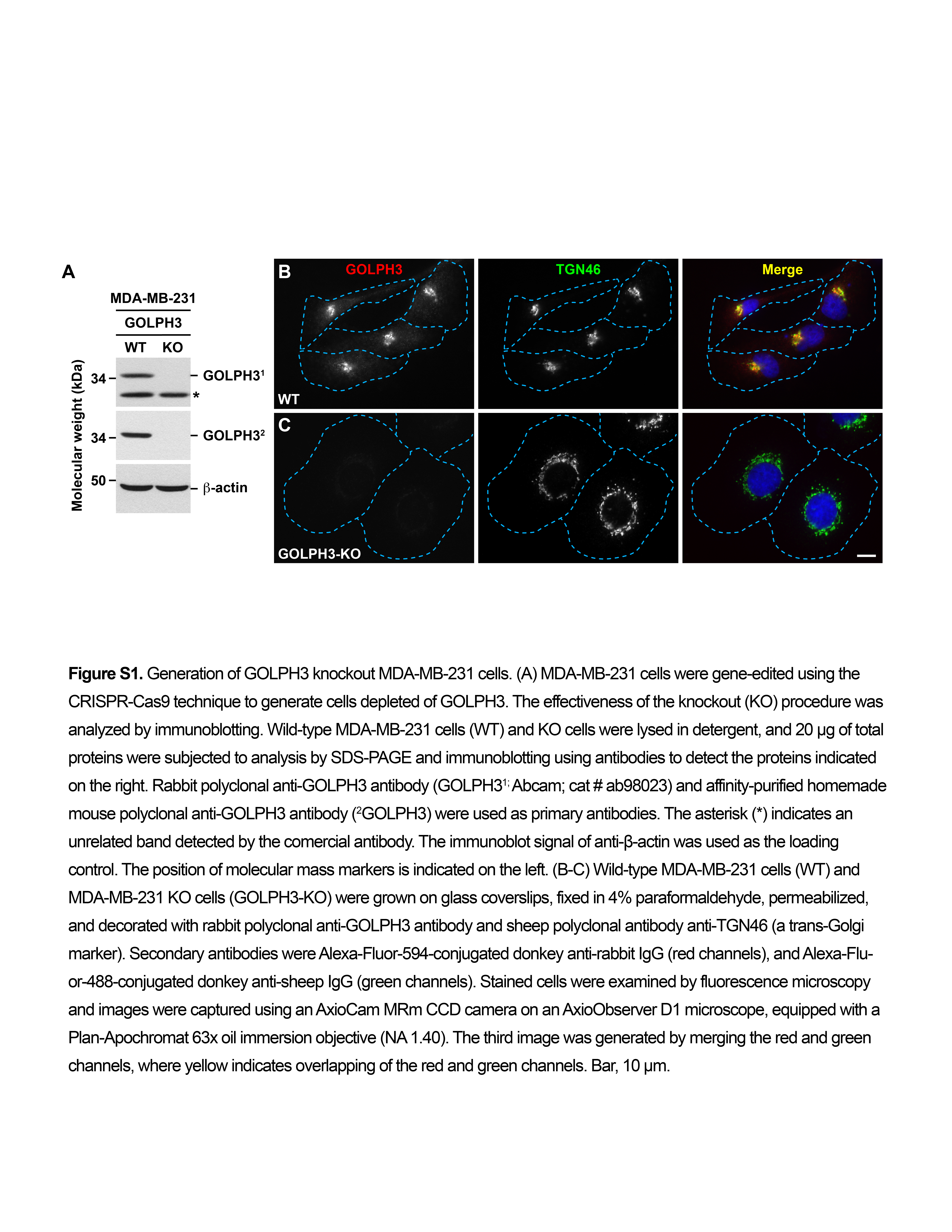

Supplement: Supplementary file 1 [file cells-13-00316-s001.zip › Fig_S1.jpg]

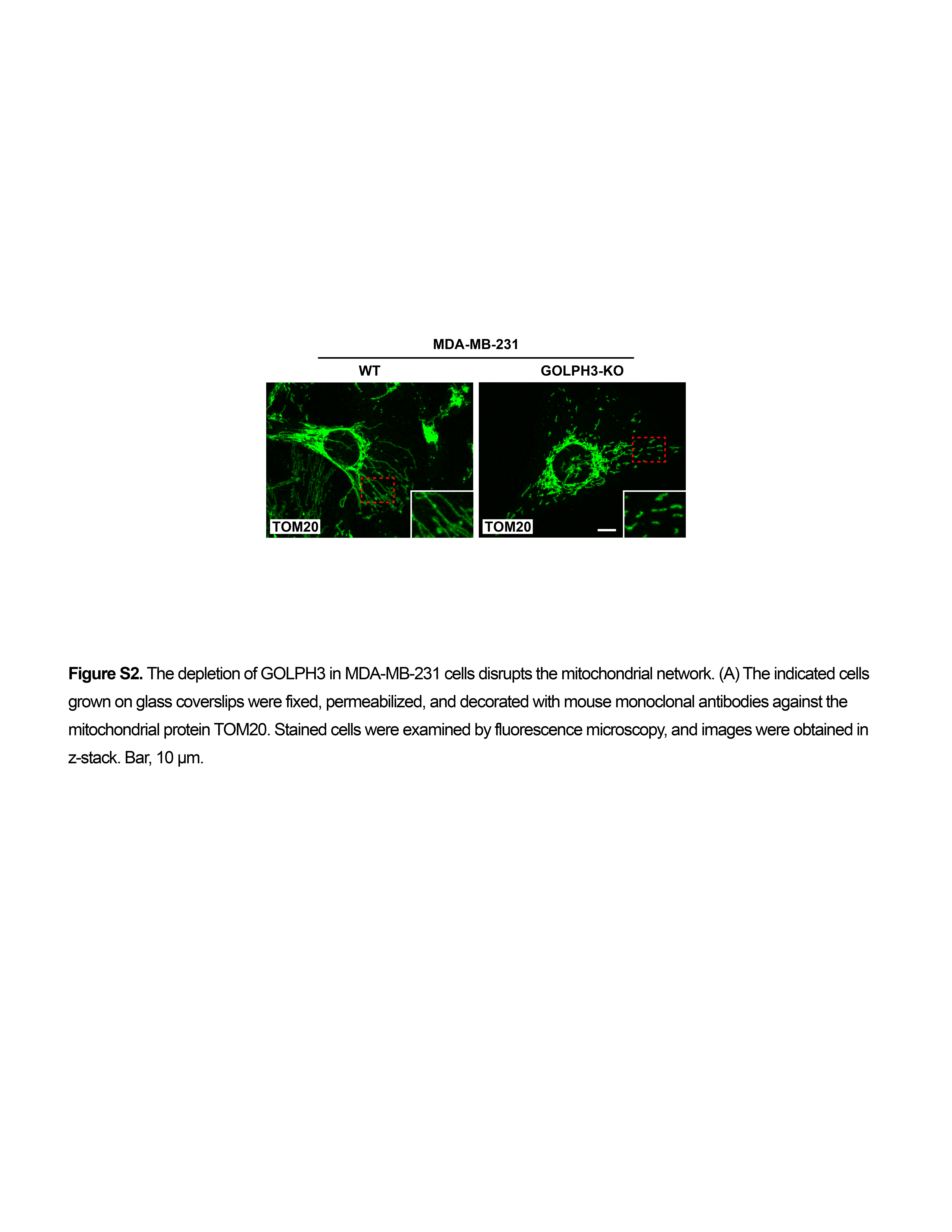

Supplement: Supplementary file 1 [file cells-13-00316-s001.zip › Fig_S2.jpg]

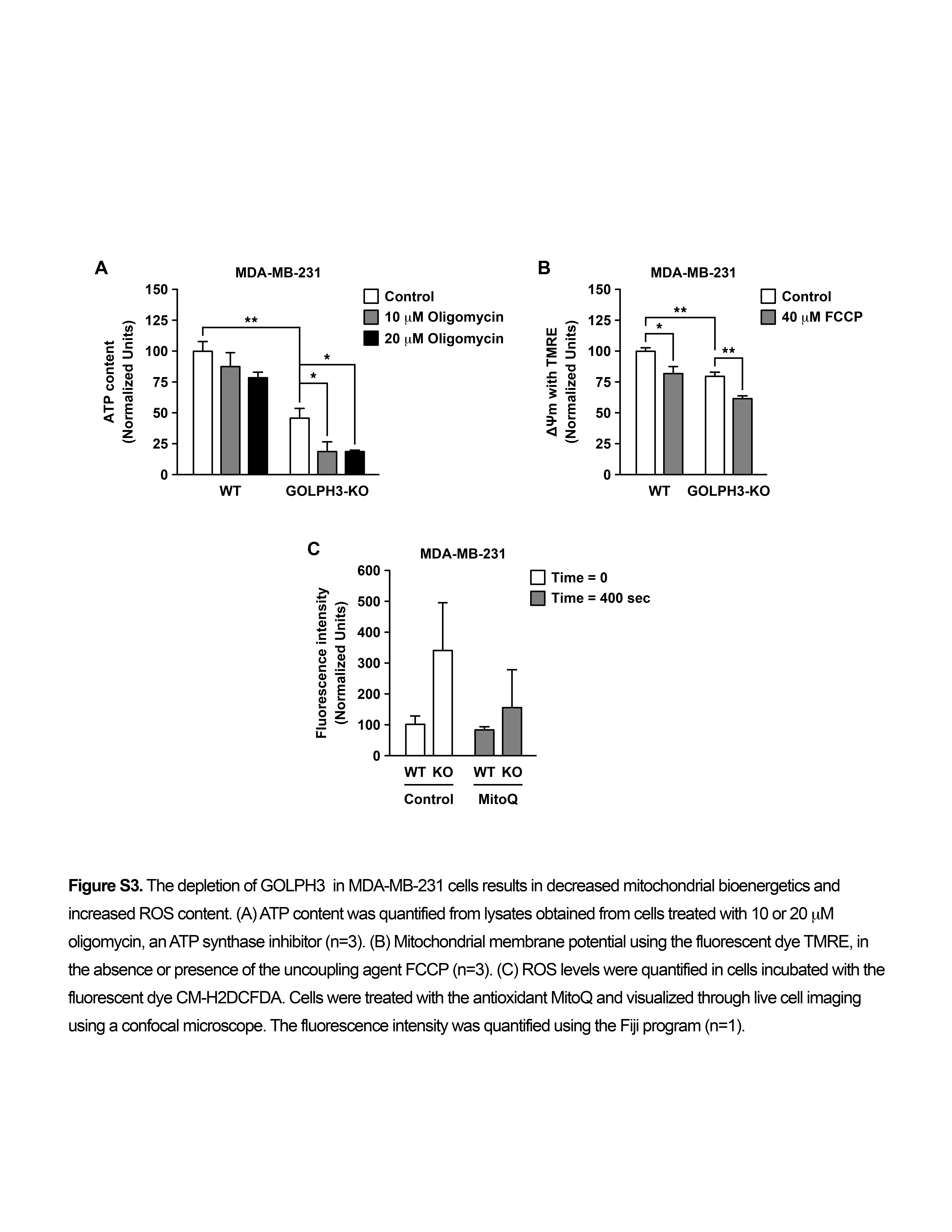

Supplement: Supplementary file 1 [file cells-13-00316-s001.zip › Fig_S3.jpg]

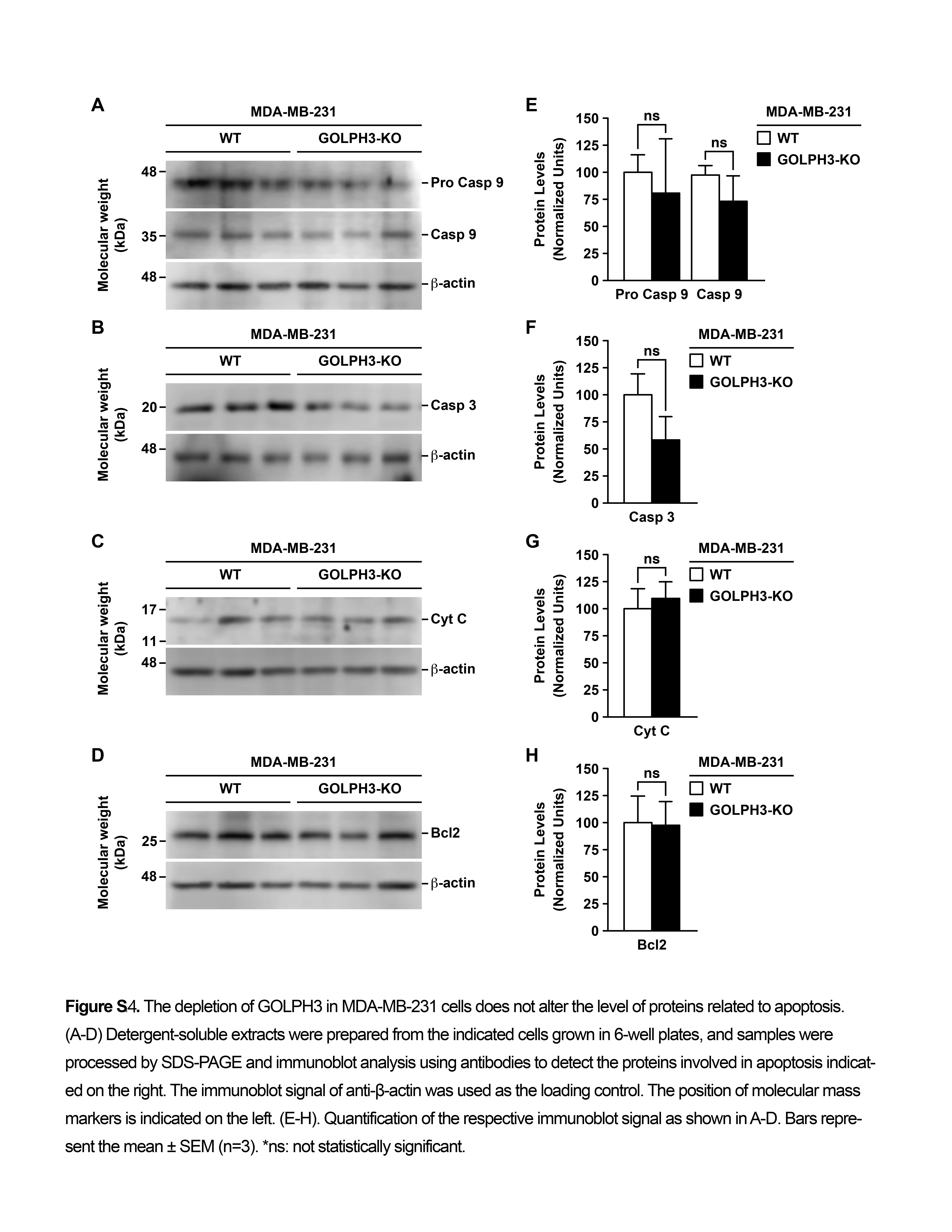

Supplement: Supplementary file 1 [file cells-13-00316-s001.zip › Fig_S4.jpg]
